# Hospital cohort study on survival predictors for intubated coronavirus disease 2019 patients

**DOI:** 10.1590/1806-9282.20231464

**Published:** 2024-05-17

**Authors:** Fabiola Jahn Deschamps, Paulo Sergio da Silva Deschamps, Laura Correa da Silva, Ellen Karkow Blos, Eduardo Schmidt Savoldi, Maria Julia Coelho Garcia, Guilherme Jönck Staub, Franciani Rodrigues da Rocha, Gabriel Zorello Laporta

**Affiliations:** 1 Centro Universitário Faculdade de Medicina do ABC, Graduate Program in Health Sciences – Santo Andre (SP), Brazil.; 2 Universidade para o Desenvolvimento do Alto Vale do Rio Itajaí, School of Medicine – Rio do Sul (SC), Brazil.; 3 Hospital of the High Itajai River Valley – Rio do Sul (SC), Brazil.

**Keywords:** Airway management, COVID-19, Critical care, Intubation, Pneumonia, Respiratory distress syndrome

## Abstract

**OBJECTIVE:**

The objective of this study was to assess the predictors of survival among patients with coronavirus disease 2019 who underwent tracheal intubation, as part of a hospital cohort study.

**METHODS:**

This retrospective cohort study in the Rio do Sul County Hospital, Santa Catarina, Brazil, from April 2020 to May 2021, focused on patients aged 18 years or older intubated for coronavirus disease 2019. We assessed the 90-day survival of intubated patients by estimating the hazard ratio using a Cox proportional hazards regression model.

**RESULTS:**

The study included 132 participants, with an average age of approximately 60 years. Tracheal intubation was successfully accomplished in 97% of cases within two attempts. The overall mortality rate was 62.9%. Notably, mortality rates were significantly higher in patients aged over 60 years (hazard ratio=2.57; 95%CI 1.54–4.29; p<0.001), those with blood oxygen saturation below 85% (hazard ratio=1.92; 95%CI 1.03–3.57; p=0.04), instances where tracheal intubation was carried out using a conventional laryngoscope (hazard ratio=2.59; 95%CI 1.22–5.48; p=0.013), and when performed by emergency physicians (hazard ratio=3.96; 95%CI 1.51–10.4; p=0.005).

**CONCLUSION:**

Our analysis unveiled that the risk of death in intubated coronavirus disease 2019 patients is four times higher when an emergency physician, as opposed to an anesthesiologist, leads the tracheal intubation team.

## INTRODUCTION

During the 2020–2021 coronavirus disease 2019 (COVID-19) pandemic, the rapid spread of severe acute respiratory syndrome coronavirus 2 (SARS-CoV-2) resulted in severe respiratory disease. Many affected areas lacked essential resources and infrastructure, leading to increased patient mortality. The initial World Health Organization data revealed hospitalization rates of 10–15%, with 39% requiring intensive care and 23% needing tracheal intubation and mechanical ventilation^1^. Despite standard protocols, global mortality of patients on mechanical ventilation ranged from 30 to 80%^2,3^.

Coronavirus disease 2019 patients who needed tracheal intubation were severely ill and at risk of cardiovascular collapse^4^. Tracheal intubation was often performed rapidly, without prior face mask ventilation to prevent contamination, leading to quick desaturation and worsened conditions. These challenges complicated COVID-19 patient tracheal intubation^5,6^.

Worldwide healthcare had to adapt protocols for dealing with COVID-19. Specialized tracheal intubation teams were formed, including the Catarina Intubation Team in the Regional Hospital of Rio do Sul County, Brazil, in March 2020. The goal of this hospital cohort study was to assess the predictors of survival among COVID-19 patients who underwent tracheal intubation.

## METHODS

This single-center cohort study occurred at High Itajai River Valley Hospital in Rio do Sul County, Brazil. The Research Ethics Committee of the University for the Development of the High Itajai River Valley granted an exemption from the requirement for patient consent forms for this observational study, which relied on data from information systems. The study received approval on May 28, 2021, with final approval n. 4741418. Eligible participants were adults (≥18 years) with positive COVID-19 tests requiring tracheal intubation from April 2020 to May 2021, excluding COVID-negative patients and those transferred to other hospitals. Survival analysis extended to 90 days from the tracheal intubation date. Our intubation team comprised 17 volunteers, with shifts covering all days of the week, including anesthesiologists, intensivists, anesthesiology residents, and emergency physicians.

Airway management adhered to a COVID-19-specific tracheal intubation protocol, guided by national and international guidelines^5-7^. All members underwent practical training in protocol implementation, airway management, and personal protective equipment use. The team followed institutional procedures, with a group established for debriefing and error correction. Both conventional laryngoscopes and videolaryngoscopes (McGRATH™ MAC—Aircraft Medical LTD., Edinburgh, UK) were employed for tracheal intubation using prepared kits with essential materials, medications, and personal protective equipment. These kits were strategically placed in hospital wards, the emergency department, and the intensive care unit (ICU) COVID-19 sector. Outcomes were documented in medical records and on a form completed by team members. Information on length of stay and survival was extracted from patients’ medical records.

To estimate hazard ratios (HR), Cox proportional hazard regression models were used for statistical analysis. In the initial phase, the model was applied to each category of explanatory factors, including demographic data (block 1), comorbidities (block 2), symptoms (block 3), and factors related to tracheal intubation (block 4). The model then adjusted each patient’s length of hospital stay based on the outcome (death or discharge) in relation to these factors. The predicted outcome provided an estimate of the risk of death in relation to the duration of hospitalization, accounting for each factor. A 5% significance level was set for two-tailed Wald tests, and statistically significant factors were incorporated into a final multiple regression model.

We scrutinized the statistically significant variables while considering sample adequacy and the statistical power provided for hypothesis testing. To calculate the required number of deaths, we used the formula suggested by Schoenfeld^8^, which considers the study’s specifics:

$$\mathrm{necessary}\;\mathrm{deaths}=\frac{{\left(z_{\beta }+z_{1-\alpha }\right)}^{2}}{\left(\mathrm{Pro}p_{E}\mathrm{Pro}p_{\mathrm{NE}}\log_{e}^{2}\Delta \right)}$$

where, to achieve a power of 80% at a one-tailed significance level of 5% (*z*_*β*_ = 0.841 and *z*_1-*α*_ = 1.645), it becomes essential to determine the minimum number of observed deaths for each group of exposed individuals (*Prop*_*E*_), unexposed individuals (*Prop*_*NE*_), and log-natural scale regression coefficient ( $\log_{e}^{2}\Delta$ ).

Complementary analyses included sensitivity, specificity, and accuracy for difficult airway prediction vs. confirmation using chi-square testing (p<0.05).

## RESULTS

A cohort of 132 participants with an average age of around 60 years was recruited for the study. The majority were male, and the predominant ethnicity was Caucasian. Prevalent comorbidities included systemic arterial hypertension, diabetes mellitus, obesity, and heart disease. Participants exhibited various symptoms, with cough, dyspnea, and fever being the most frequently reported (Table 1).

**Table 1:** Coronavirus disease 2019 patient data, Rio do Sul County, Brazil, April 2020 to May 2021.

Demographic data, comorbidities, and symptoms	Mean (SD), median (IQR), or n (%)
	(n=132)
Age	Mean 59 (SD 13)
Sex	
Male	79 (59.8%)
Female	53 (40.2%)
Race	
White	126 (95.5%)
Other	6 (4.5%)
Comorbidities	117 (88.6%)
Obesity	44 (33.3%)
Systemic arterial hypertension	78 (59.1%)
Diabetes mellitus	46 (34.8%)
Thyroid disease	9 (6.8%)
Chronic obstructive pulmonary disease	8 (6.1%)
Asthma	7 (5.3%)
Heart disease	17 (12.9%)
Nephropathy	5 (3.8%)
Neurological disease	10 (7.6%)
Psychiatric disease	11 (8.3%)
Cancer	8 (6.1%)
Other	41 (31.3%)
Symptoms	
Dyspnea	88 (66.7%)
Cough	100 (75.8%)
Fever	62 (47%)
Coryza	25 (18.9%)
Odynophagia	13 (9.8%)
Fatigue	57 (43.2%)
Myalgia	48 (36.4%)
Smell or taste disorder	16 (12.1%)
Chest pain	3 (2.3%)
Headache	28 (21.2%)
Gastrointestinal disorder	19 (14.4%)
Other	5 (3.8%)
O_2_ saturation (SaO_2_)	
<85%	36 (27.3%)
85–90%	53 (40.2%)
>90%	43 (32.6%)
Days of symptoms	Median 11 (IQR 7)

All 132 patients experienced respiratory failure requiring tracheal intubation. Successful intubation rates were 91.7% on the first attempt and 97% on the second attempt. Predictions of difficult airways were made in 14.4% and confirmed in 8.3%, with moderate sensitivity (55%) and high specificity (89%). The prediction’s accuracy was 86% (p=0.0004), highlighting its reliability in anticipating difficult airways. However, the study’s unfortunate high mortality rate was evident in the 90-day follow-up, with 83 deaths (62.9%) and 49 discharges indicating recovery (27.1%). For further insights, Table 2 details the factors associated with tracheal intubation.

**Table 2:** Tracheal intubation associated data, Rio do Sul County, Brazil, April 2020 to May 2021.

Factors related to tracheal intubation	n (%)
Intubation team leader	
Anesthesiologist	93 (70.5%)
Intensivist	9 (6.8%)
Emergency physician	9 (6.8%)
Anesthesiology resident	21 (15.9%)
Intubation environment	
Emergency department	85 (64.4%)
Intensive care unit	47 (35.6%)
Number attempt count	
1	121 (91.7%)
2	7 (5.3%)
3	3 (2.3%)
4 or more	1 (0.8%)
Support staff	
Physician	62 (47%)
Nurse	64 (48.5%)
Nursing technician	4 (3%)
Physiotherapist	2 (1.5%)
Pharmaceuticals used in intubation	
Ketamine + rocuronium	108 (81.8%)
Etomidate + alfentanil + rocuronium	18 (13.6%)
Etomidate + rocuronium	6 (4.5%)
Laryngoscope variety	
Videolaryngoscope	20 (15.2%)
Conventional laryngoscope	112 (84.8%)

In our analysis of adjusted Cox proportional hazards regression models, we observed a fourfold increase in the risk of death when tracheal intubation was performed by emergency physicians (HR=3.96, 95%CI 1.51–10.4; p=0.005), with similar findings for conventional laryngoscope use (HR=2.59, 95%CI 1.22–5.48; p=0.013), patient age over 60 years (HR=2.57, 95%CI 1.54–4.29; p<0.001), and severe hypoxemia (HR=1.92, 95%CI 1.03–3.57; p=0.04). These results maintain statistical power due to sufficient sample size (Table 3).

**Table 3:** Factors linked to coronavirus disease 2019 mortality post-intubation as derived from Cox proportional hazards models with adjustments, Rio do Sul County, Brazil, April 2020 to May 2021.

	Adjusted hazard ratios (95%CI)	p (Wald’s test)	Adequate sample size—80% power and 95% significance level
Intubation environment			
Intensive care unit			
Emergency department	1.74 (0.99–3.07)	0.055	No
Intubation team leader			
Anesthesiologist			
Intensivist	0.97 (0.33–2.89)	0.963	
Anesthesiology resident	0.93 (0.45–1.95)	0.852	
Emergency physicians	3.96 (1.51–10.4)	0.005	Yes
Laryngoscope variety			
Videolaryngoscope			
Conventional laryngoscope	2.59 (1.22–5.48)	0.013	Yes
Neurological disease	1.96 (0.88–4.41)	0.102	No
Age			
≤60 years			
>60 years	2.57 (1.54–4.29)	<0.001	Yes
O_2_ saturation			
>95%			
85–90%	0.94 (0.51–1.75)	0.857	
<85%	1.92 (1.03–3.57)	0.04	Yes

## Discussion

In this context, our performance met expectations, showcasing a high success rate within one or two attempts for tracheal intubations. The research reveals a moderate sensitivity and noteworthy specificity, underscoring the substantial accuracy attainable through difficult airway prediction. The study emphasizes advanced age and severe hypoxemia as independent and cumulative mortality factors in COVID-19 patients. Moreover, our findings suggest reduced survival rates among patients intubated by emergency physicians.

The surge in COVID-19-related respiratory failure has strained global healthcare providers. Ensuring successful, efficient endotracheal intubation is crucial, with specialized teams offering better outcomes. Patient survival depends on factors such as age and pathology severity, while equipment and clinician skill also influence results. Intubation teams have demonstrated proficiency in COVID-19 patient intubations, achieving initial success rates between 85 and 92%, which increased from 97 to 98% upon the second attempt^9-11^. In their study on COVID-19 patients, Zheng et al. reported intubation success rates using videolaryngoscopes ranging from 89 to 94% on the first attempt and 80 to 100% on the second attempt^12^. Lower success rates were observed with conventional laryngoscope, at 70% on the first attempt and 83% on the second attempt^12^. Conversely, Wong et al. observed no variation in intubation success rates on the first attempt between the two equipment types^13^.

The American Society of Anesthesiologists’ guidelines advise conducting a pre-airway management risk assessment for difficult airway situations^14^. In a meta-analysis of 50,760 patients, the prediction of a difficult airway demonstrated low to moderate sensitivity (20–62%) and moderate to high specificity (82–97)^15^. Similarly, Norskow et al. observed a notably higher specificity compared to sensitivity in difficult airway prediction^16^.

Several studies have consistently reported increased mortality among elderly COVID-19 patients with severe hypoxemia^17-21^. Increased mortality among older patients can be linked to age-associated immune responses, culminating in reduced effectiveness and heightened inflammation^19,21^. Hypoxemia is intricately tied to an inflammatory response. Patients afflicted by severe hypoxemia in the context of COVID-19 exhibit elevated levels of proinflammatory cytokines, lung injury, and acute respiratory distress syndrome^20,21^. Tang et al. observed higher mortality with intubations by emergency physicians compared with anesthesiologists^22^.

This study has notable limitations, including its observational nature, absence of a comparator group, and being conducted at a single center.

## CONCLUSION

In our study, mortality was four times higher when tracheal intubation was conducted by emergency physicians compared with cases where an anesthesiologist served as the intubation team leader. This adjustment accounts for confounding factors, including intubation location, comorbidities, patient age, and disease severity.
